# First-person accounts of the processes and planning involved in a suicide attempt on the railway

**DOI:** 10.1192/bjo.2020.173

**Published:** 2021-01-20

**Authors:** Ian Marsh, Lisa Marzano, David Mosse, Jay-Marie Mackenzie

**Affiliations:** Faculty of Medicine, Health and Social Care, Canterbury Christ Church University, UK; Department of Psychology, Middlesex University, UK; Department of Anthropology and Sociology, School of Oriental and African Studies University of London, UK; Department of Psychology, University of Westminster, UK

**Keywords:** Suicide, railways, suicidal process, first-person accounts

## Abstract

**Background:**

The processes and planning involved in choosing and attempting to die by a particular method of suicide are not well understood. Accounts from those who have thought about or attempted suicide using a specific method might allow us to better understand the ways in which people come to think about, plan and enact a suicide attempt.

**Aims:**

To understand from first-person accounts the processes and planning involved in a suicide attempt on the railway.

**Method:**

Thematic analysis was conducted of qualitative interviews (*N* = 34) undertaken with individuals who had contemplated or attempted suicide by train.

**Results:**

Participants explained how they decided upon a particular method, time and place for a suicide attempt. Plans were described as being contingent on a number of elements (including the likelihood of being seen or interrupted), rather than being fixed in advance. Participants mentally rehearsed and evaluated a particular method, which would sometimes involve imagining in detail what would happen before, during and after an attempt. The extent to which this involved others (train drivers, partners, friends) was striking.

**Conclusions:**

By giving people free reign to describe in their own words the processes they went through in planning and undertaking a suicide attempt, and by not interpreting such accounts through a lens of deficit and pathology, we can arrive at important insights into how people come to think and feel about, plan and enact a suicide attempt. The findings have implications in terms of understanding suicide risk and prevention more broadly.

In the UK in 2018, there were 6507 suicides, 686 more than in 2017, representing a statistically significant increase in the suicide rate, with 11.2 deaths per 100 000 population in 2018 compared with 10.1 deaths per 100 000 population in 2017.^1^ On the UK railway network there were 302 suicide or suspected suicide fatalities (271 on mainline railways and 31 on London Underground) in 2018–2019,^2^ which equates to around 4.5% of all suicides. Although a small percentage of the total number of suicides, such deaths on the railways can have devastating consequences for families, the rail industry, staff dealing with the aftermath of such incidents and potential witnesses.

Previous studies have examined the factors involved in the decision to choose a particular method of suicide (e.g. hanging,^3^ firearms,^4^ falling from height^5^ and the railways^6^), as well as exploring the different sources of information accessed by people contemplating ending their lives, including the internet^7–10^ and the media more broadly.^11^ Work has also been undertaken on how the choice of methods relates to variables such as gender,^12–14^ age^15,16^ and psychiatric diagnosis,^17,18^ and how the method choice can change over time.^19,20^

Recent studies have also attempted to cast light on the factors and processes involved in moving from choosing a particular method to planning and then enacting an attempt.^21–25^ Traditional models of suicide, it has been argued, are poor at distinguishing those who experience thoughts of suicide but do not act on them from those who do make an attempt to end their lives.^26^ The development of ‘ideation-to-action’ models has sought to overcome these limitations.^27,28^ As Nock et al argue, ‘[w]e need to better understand how people move along the entire pathway to suicide: from onset of the thought, to developing a plan and intention, to making preparations, to making a decision to act, and actually carrying out the attempt’.^29^

People with lived experience of suicide attempts can provide valuable insights into how plans for suicide come to be formulated, planned and carried out. In particular, accounts from those who have thought about or attempted suicide using a specific method might allow us to better understand the process of moving from the planning to the enactment stage.

## Method

### Study design

As part of a wider study of suicide on the railways, we conducted qualitative interviews to explore in depth the lived experiences of those considering and/or attempting suicide by train. Unless otherwise specified, this included suicidal thoughts and behaviours involving walking, jumping or lying in front of a train (at stations, level crossings, railway bridges and on open tracks, on both mainline/overground and metro/underground networks).

### Interview participants

We interviewed UK-based men and women aged 18 years or over who had: survived a suicide attempt on the railways (‘group A’); survived a suicide attempt by another method, having considered but rejected a rail suicide (‘group B’); or experienced thoughts of rail suicide but not made a suicide attempt (‘group C’). Recruitment occurred via a wider online survey (see Marzano et al^6^ for further details) and the British Transport Police (BTP) (subject to a privacy impact assessment, eligible group A participants were sent a brief letter about the research by BTP).

### Materials and procedures

A semi-structured interview schedule was used to explore participants’ experiences of suicidality on the railways, including perceived triggers and motivations, as well as barriers against railway suicide which influenced their decision-making (where applicable). Interviews with group A and B participants included specific questions about the factors, circumstances and processes that led up to their rail-related suicidal thoughts and/or behaviour, including their reasons for the specific timing and location of their attempt, and the precautions taken (if any) while planning or preparing for it.

All interviews were conducted by an experienced suicide researcher, following written informed consent. Some group A interviews were conducted face to face in a private room at a local Samaritans^a^ branch or at the researchers’ universities (*n* = 6). Others were carried out by telephone (*n* = 17) or email (*n* = 11, including all group C interviews).

### Analysis

All interviews were taped, transcribed verbatim and anonymised. The first phase of the analysis involved an inductive thematic analysis (following the six key stages recommended by Braun and Clarke^30^) to identify the range of factors deterring and prompting the decision to attempt suicide on the railway networks and the behaviours immediately preceding an attempt/planned attempt at a station. The results of these analyses are presented elsewhere (see, respectively, Marzano et al^11^ and Mackenzie et al^31^).

Whereas the initial analysis focused on why individuals consider, use or disregard a rail suicide method, and the factors which might influence their decision, the second phase of the analysis (discussed here) involved re-examining the interview data, focusing on and exploring in more detail first-person descriptions of the processes and planning involved in considering and making a suicide attempt. Although there is, inevitably, some overlap between these two stages of analysis, the guiding idea was an attempt to supplement the analysis of why people choose or disregard the railway as a method with a wider exploration of how people plan and (where applicable) carry out a suicide attempt, and to cast light on the processes involved in, and experiences of, the movement from thought to action. In Nock et al's^29^ terms, we have tried here to consider the ‘pathway to suicide’ from the development of a plan to the preparations undertaken and, for some, the carrying out of an attempt.

Analysis involved an iterative process of open, axial and selective coding. In the initial stage of open coding, interview transcripts were coded in a way that captured the thoughts, ideas and statements of each interviewee. The transcripts were then reviewed in the second stage of analysis (axial coding); here, broader themes were assigned to posts that consolidated the open codes. In the third and final stage of analysis, selective codes were identified that represented the central or main themes under investigation (i.e. how people come to think about, plan and enact a suicide attempt).

Although it would have been valuable to make comparisons between participant groups, this proved difficult to do in a meaningful way owing to the relatively small numbers involved.

### Ethics

The authors assert that all procedures contributing to this work comply with the ethical standards of the relevant national and institutional committees on human experimentation and with the Helsinki Declaration of 1975, as revised in 2008. All procedures involving human subjects were approved by the Psychology Department Research Ethics Committee at Middlesex University (ethical approval reference: ST019-2015). Written informed consent was obtained from all participants.

## Results

We conducted 34 in-depth interviews: 10 with group A participants (7 males and 3 females), 14 with group B (3 males and 11 females) and 10 with group C (2 males and 8 females). Participants’ ages ranged from 18 to 72 years, with the majority describing themselves as White British (with the exception of two British Indian, one Irish, one ‘mixed’, one Arab, one European and one US-born participant).

## Main themes

### Choosing a site/method of suicide

Study participants described an active, dynamic process in terms of choosing a particular method, time and place for a suicide attempt, and in terms of the actions they took towards that goal. They described their plans as being contingent on a number of elements, rather than being something entirely fixed in advance.

Often these were framed in terms of necessary or desirable elements for a particular method or place: how long it would take to die (quick/slow); the perception of how likely a method was to end their life (lethality); the amount of pain likely to be suffered, and for how long; and the likelihood of being seen, interrupted or stopped. Other factors included whether the site was considered private or public, and the likely state and site of their body afterwards.

One interviewee (B1) was quite explicit about the different criteria he used when choosing a particular method of suicide:

‘*I've come up with, for me, 5 criteria. Of why I did it. And the first criteria was ease of equipment or ease getting to a location… so the location and getting hold of the stuff… one of the other criteria was the probability of success once I started the attempt… duration and intensity of pain during the attempt… top criteria really was that I didn't want my body found by my wife.’* [survived a suicide attempt by another method, having considered but rejected a rail suicide]

### Choosing railways as a site/method of suicide

When considering the railways as a particular means and/or place to end their life, participants identified a number of common considerations.

#### Lethality^b^

Railway suicide was described as being a highly lethal method, one that was likely to be fatal:

B7 *‘I thought it would be definite for sure, because trains when they're coming really quickly, they're not going to stop. That's why I think it's one of the most easy, quick methods, because it's just going to hit you.’* [survived a suicide attempt by another method, having considered but rejected a rail suicide]

Cx *‘part of what has always stopped me attempting suicide is the chance that I might not die but with railway suicides survival is highly unlikely.’* [experienced thoughts of rail suicide but not made a suicide attempt]

#### Efficiency

It was seen as a method that is likely to be quick:

A1 *‘A quick, violent death is quite attractive. I think that's one thing that you hope that a train can provide.’* [survived a suicide attempt on the railways]

Cx ‘*The force from a train would be enough for it to be an instant death so I would feel no pain, and for no one else to be able to find what was me would help others get over the fact that I wouldn't be back.*’ [experienced thoughts of rail suicide but not made a suicide attempt]

#### Accessibility and privacy

As well as being perceived as a ‘reliable’ and quick way to end your life, the railways were also taken to be an accessible and ‘anonymous’ location for suicide:

A1 ‘*the great thing about the train stations is partly the sense of anonymity, whether it's train stations or open stretches of track… It was essentially no one could see you and that was quite practical.’* [survived a suicide attempt on the railways]

*A4 ‘I'd want to do it somewhere privately… It's not the sort of thing you want to do in front of everybody for a show.’* [survived a suicide attempt on the railways]

#### The effect on others

One of the most consistent features of the interviews was the extent to which participants highlighted how a consideration for others informed their decision-making in relation to their choice of method, and the time and place of their attempt. The most prominent concern expressed about suicide on the railways was the effect it would have on others, particularly the train driver. There was also concern expressed about family members having to identify the body:

A4 ‘*I feel desperately sorry for the person who was driving that train and other people that may have witnessed it and how that's going to affect them because it will*.’ [survived a suicide attempt on the railways]

B4 ‘*I couldn't do that, because that would be running the risk of doing that to the driver of the train, and likewise a car, I couldn't do that, because that's making somebody else complicit, so that's almost making them feel as if they'd killed me.’* [survived a suicide attempt by another method, having considered but rejected a rail suicide]

#### Possibility of surviving with injuries

The possibility of surviving with injuries was often acknowledged, with participants describing giving consideration to whether they might survive the attempt, and what that survival would be like, imagining or visualising it in some detail:

A1 ‘*what I don't want to be is in some sort of half vegetative state. That just seems the worst of all worlds. Your current life may be a bit ropey from time to time, but it's definitely got to be better than being a bit of a physical wreck or a mental wreck.’* [survived a suicide attempt on the railways]

B10 *‘if you've got a slow train again, I don't know, I think being incapacitated afterwards or it not ending as you would expect would put me off as well.’* [survived a suicide attempt by another method, having considered but rejected a rail suicide]

*Cx ‘Because it would be almost 100% guaranteed to be totally effective, and I was terrified at the thought of pain or ending up disabled, or of causing a lot of suffering to other people.’* [experienced thoughts of rail suicide but not made a suicide attempt]

### Imagining suicide

Interestingly, participants described a process by which they imagined (or rehearsed in their mind, or even in practice) and evaluated a particular method or scenario. This process seems to have involved (for some) images of travelling to a particular place; imagining the attempt itself (for example, being hit by a train), and the sorts of pain which might be involved, how long they would be alive for and so on; and imagining the scene immediately after the event – what happens to their body afterwards, the immediate effect on others (e.g. the train driver) and the effects on others later (when family would be informed, for example).

Thus, participants described having not just thoughts about a particular method but of imagining in detail what would happen to them and others before, during and after an attempt. The extent to which this involved others (drivers, partners, friends) was quite striking. Participants offered descriptions of imagining the reactions of others (e.g. to finding their body), often alongside a moral accounting for the act (in many cases, this involved assessing the likely effects on others):

A8 *‘I was like, “My partner is going to be so disappointed if I do this”. Because like he said, I'd been trying hard over the past year… To like stay well… and he was at home during the time. At that time. And I was imagining the kind of call he would get. And like there have been a lot of times this year when just on an impulse, I've had a bad day at work, and I'm like walking down to the platform, I can hear a train coming so I walk a little faster towards the platform and I'm a little… and every time a train… I'm on the platform, I actually have to step back.’* [survived a suicide attempt on the railways]

The suicidal act had seemingly often been already rehearsed (in mind or practice) – it didn't just exist as an abstract thought or ‘ideation’. Detailed narratives were sometimes constructed around the event that had structure, characters (self and other), and environments, and were associated with meanings and images that were affectively charged:

A8 *‘But with like, I guess, creating a suicide, you're creating a narrative that's special and meaningful to you, and… and it was the thought of, I guess, doing something right for once. And like making it a spectacle and making it, I guess, theatrical. I even had like… I'd created a playlist. To kind of accompany it. So it had like lots of… lots of… mostly classical music. Like kind of Beethoven and Puccini. Theatrical stuff. And then when I was considering the railway, when I wasn't actually able to go through with it in the end, again it was a kind of last resort, I guess.’* [survived a suicide attempt on the railways]

For others, the plans were less clearly outlined but had still been contemplated for a long time:

A7 ‘*I sort of like had a sketchy plan and it's always been in my mind as an option, so that's a sort of plan but in terms of like that one specifically and – no, I just sort of had it in my head that that's – let's sort of do this. Let's consider a way and I think I was just cycling along until I found somewhere I thought was suitable where someone was unlikely to find me.’* [survived a suicide attempt on the railways]

#### Person's relationship to suicidal thoughts and imaginings

There was sometimes a marked difference in the relationship to suicidal thoughts, ranging from someone finding them ‘terrifying’ to another reporting that they ‘calmed down’ once they thought of suicide:

Cx ‘*the main thing about suicidal thoughts is that they are completely and utterly terrifying.*’ [experienced thoughts of rail suicide but not made a suicide attempt]

B1 ‘… *I just couldn't sleep. It was impossible, every waking second I had these thoughts racing around my head and I just couldn't stop it. I was on the internet, I was trying to figure out what was going on, couldn't sleep and I suddenly thought about suicide. And my mind calmed down. That was the only way that I could describe it. Absolutely calmed down, and I went on the internet and started to research suicide methods basically.*’ [survived a suicide attempt by another method, having considered but rejected a rail suicide]

### Personal meaning of railway places

The interviews often connected railway spaces to certain personal (but possibly culturally shared) ideas, feelings or associations related to the rail network as a site for attempted suicide. Some expressed strong negative associations with the railways that made them the right place for the ending of life:

A1 ‘*I do a lot of public transport and I've been delayed by other people's suicide and one has this kind of grudge against the train system here…*’ [survived a suicide attempt on the railways]

Cx ‘*I personally have always wanted to work for rail, and been finding it hard to break into the industry, so at some points I felt like it would be like a (for a lack of a better phrase) last ‘fuck you’ to the industry for not considering me*.’ [experienced thoughts of rail suicide but not made a suicide attempt]

For some, the high adrenaline experience of the ‘rushing and hammering’ of trains was connected to energy and impulsivity. For others, death on the railways would involve a kind of violence and punishment:

A3 ‘*As I say, you know, it was the idea of physically smashing myself up, I think. … it was like rejection. So that – this was triggering a bit of – sort of self-doubt and self-hate. On a physical level. And I think, you know, it was almost the idea of physically destroying myself was quite cathartic, in a way.’* [survived a suicide attempt on the railways]

Sometimes railways were chosen because of negative associations, and the places considered for a suicide attempt were sites of traumatic personal experience. Alternatively, the railways were spoken of as non-places, anonymous, depersonalised and away from significant others:

Cx ‘*I found that whenever I was passing through a train station (generally for business journeys on my own) suicidal thoughts would become stronger. I believe that the busy nature of the stations and lack of personal contact with anyone made this easier to contemplate*.’ [experienced thoughts of rail suicide but not made a suicide attempt]

In some cases, the railways (or underground) were sites of prior experience or involved knowledge of train speeds, frequency, access (open stretches), the height of fences or bridges, or unobservability. These sites might be visited to establish these facts or to test the idea of a suicidal act (e.g. by standing on a bridge). The salience of the railways is here reported in practical terms:

A6 ‘*I used to spend a lot of time on the Underground. And it was then I really started thinking about the railways as a place for suicide, and so that was one of the links for me.’* [survived a suicide attempt on the railways]

### Enacting plans

When recounting how they went about enacting their plans, participants described engaging in an ongoing, active, dynamic appraisal of their environments. This process seemed to be fluid and contingent, with the person engaged in an ongoing interpretation and appraisal of their environment during the lead-up to an attempt.

#### Avoiding being seen

For some there was a need to avoid being seen, either because they did not want to be stopped in their attempt, or because they did not want their attempt to be witnessed:

A7 ‘*I can remember actually seeing the camera, or what I thought was a camera on the railway. I didn't actually see the camera itself, I seen what looked like an infra-red illuminator, which they use for like night vision. So it's basically like a giant arch giving out infra-red but we can't see it but like the cameras can and I was seeing like the red blob that looked like it was an infra-red illuminator for a camera so I made sure I was out of the way of that.’* [survived a suicide attempt on the railways]

B1 ‘*I can remember in my mind thinking that I didn't want anybody to see me when I was going to kill myself. So I don't know if I'd have gone to the car park if there was people around, would I have gone through with the attempt at that moment in time. I don't know, I was very keen not to be seen or not to interfere with people. That's another reason why I don't think I would have chosen the railway station. Too many people about*.’ [survived a suicide attempt by another method, having considered but rejected a rail suicide]

#### Avoiding interventions or interference

Often people were quite explicit about the lengths they went to avoid being intervened with. For some this was to do with being arrested and possibly admitted to hospital; for others it was more to do with wanting their ‘own space’:

A6 ‘*I was very frightened of being stopped so I was trying to look normal and look like I had some purpose when actually I went down to do it so that nobody would stop me because I didn't want there to be any chance of getting stopped and arrested again so I was very clear that once I did it I would just do it, there would be no pause on the edge or anything*.’ [survived a suicide attempt on the railways]

A7 ‘*just by the location and moving away so that – yeah, so that people didn't find me; I didn't want any intervention – it was sort of that's what I'm going to do and I want my own space before I do it.’* [survived a suicide attempt on the railways]

Cx *‘I had been aware that other stations there were signs saying to approach a member of staff if you were struggling.… I couldn't see anyone to approach. I was terrified of what they would do if I did say something. I presumed that police would be called, that I may be hospitalised in a strange city far from friends and family.’* [experienced thoughts of rail suicide but not made a suicide attempt]

#### Just prior to attempt

For participants who had made a suicide attempt, their descriptions of events often included recollections of their emotions at the time:

A5 ‘*They knew that I was having issues because I was shouting and throwing stuff about in my room and I've never done anything like it before, I was just going by feelings and thoughts. So I didn't say, I don't remember saying, but they knew that I was heading towards the train station, so that's why they called, because I think they assumed the worst*.’ [survived a suicide attempt on the railways]

A6 ‘*I was very frightened because I was scared of the police from previous experiences, it was a horrible, horrible experience. And I got more and more frightened and I started to make my way to this bridge*’ [survived a suicide attempt on the railways]

#### Being interrupted and ending attempt

On occasions, it was an unexpected interruption that ended an attempt:

A3 ‘*I remember I was hanging around by the front of the tunnel and a mother and kid came in. And the kid would have been about seven years old. The kid doesn't need to see that. So that stopped me.’* [survived a suicide attempt on the railways]

B6 *‘I was going on the tube and thinking I'm going to jump but it was more like I was tired, and I just wanted this situation to end, and I felt like my body was going to jump. And I heard a baby screaming, that's what stopped me.’* [survived a suicide attempt by another method, having considered but rejected a rail suicide]

### Accidents, fate and agency

Perhaps unsurprisingly, in interviews with survivors of suicide attempts an ambivalence surrounded their intentions and agency. Some spoke of there being no alternative, no choice, others that suicide was a response to feeling out of control, or of taking the control that had been removed from them by mental health services. Several accounts implied a wish for agency to come from elsewhere, and in some the person described putting themselves in a situation where their fate would be left to chance, to impulse, or to people around who might or might not intervene:

A3 ‘*But then I decided eventually to leave it to fate. So I – I sort of lay down and went to sleep. And I was thinking, you know, there was probably that much room, the safety fence, sheer drop, and this bit that I was lying on.’* [survived a suicide attempt on the railways]

Sometimes, the attraction of an attempt on the railways was that it could, possibly, be taken by others to be an accidental death:

A3 ‘*I remember telling someone I kind of wanted it to look like an accident.’* [survived a suicide attempt on the railways]

## Discussion

Previously published research on choice of method has tended to focus on the factors people take into account when considering how to end their lives (i.e. which method to choose), and the information sources drawn on when making this decision. The interviews in this study also explored the later stages of the ‘suicidal process’, that is from the development of a plan, to the preparations undertaken, the decision to act and, in some cases, the carrying out of the attempt. As such, they help to illuminate the processes involved in, and experiences of, the movement from thought to action.

The findings presented here suggest that suicidal individuals are often engaged in a complex activity or set of tasks involving planning, choice, visualisation and adaptation. People recounted that they imagined and rehearsed in their minds particular scenarios involving their own death and the effect it would have on others, often constructing a detailed story about their suicide attempt. Participants also described a dynamic, iterative relationship between themselves and their social and physical environments before, during and after an attempt.

Of particular interest were the degrees of imagination and resourcefulness described by participants. Traditional approaches to understanding suicidal behaviour have tended to frame suicidal thoughts and attempts as indicative or symptomatic of some form of pathology or deficit, and, perhaps as a consequence, thoughts of suicide and planning how to end one's life are often read as arising from limited or constricted individual thought processes and reasoning. Previous research has suggested that people contemplating and planning suicide have ‘increased cognitive rigidity’ and a ‘reduced potential to plan the suicidal act in a way that maintains the possibility to adapt one's actions to changing circumstances’^32^; ‘[i]mpaired decision making’ is also highlighted, alongside deficiencies in the ‘ability to imagine the future and to engage in effective problem solving’.^33^

However, the danger in understanding suicidal thoughts and behaviours as arising primarily or solely from psychological deficits or dysfunction is that this can lead to an unnecessarily narrow (and potentially distorted) view of the processes and planning involved in a suicide attempt, with elements of resourcefulness and personal meaning potentially overlooked. This has particular consequences for how we conceptualise and theorise the movement from ‘ideation-to-action’ and our understanding of how people actually plan, prepare for and undertake a suicide attempt.

Suicide, in the accounts given by the study participants, is conceptualised as the end result of a series of actions requiring planning, decision-making and choices, and adaptability. It is a process whose beginnings often stretch back a considerable length of time. Whereas existing academic and professional accounts focus on particular cognitive aspects and states of the suicidal person, the first-person accounts summarised here construct suicide much more as a physical and logistical problem, almost as a project requiring careful planning and forethought to be ‘successfully’ completed. The different activities or tasks engaged in by participants to end their life were often infused with personal meaning, with the choice of method, time and location influenced by symbolic associations, memories and consideration for others (for example, their family or the train driver). In addition, participants sometimes described their actions before and during attempts in terms of constructing a story of sorts, one that was ‘special and meaningful’ to the person. While fully recognising that distress and its alleviation are most often the main driver behind a desire to die, the contemporaneous and personally meaningful narrating, the use of imagination and visualisation in the planning of a suicide attempt, and the degree of consideration often given to the experiences of others are less suggestive of ‘cognitive rigidity’ and ‘impairment’, more of people engaging in focused, meaningful performances of specific goal-oriented tasks.

### Limitations

The accounts presented here are from people who have survived or reconsidered a railway attempt, and the processes and planning involved in a fatal attempt could be in some important ways be different. As with all research on suicide, it is not possible to generalise from one group to another with any certainty, and this should be considered an important context in relation to the analysis presented here. As an example, it is possible that the careful planning and forethought described by the study participants may be reflective, in part, of a degree of uncertainty or ambivalence about going ahead with a suicidal act, which may be different from those who complete suicide.

It is also necessary to acknowledge that the study participants’ stories were told from the perspective of the present, that is, they are reconstructions of events. They do not provide unmediated access to ‘what really happened’ but are accounts given in another context and as such can involve elements of moral accounting and positioning intended to lessen perceived or anticipated criticism from others for their actions. It is worth noting that interviewees may well have been asked to recount their story to numerous other people (e.g. emergency workers, doctors, nurses, therapists, families, friends and so on), and through this process a version of events may have evolved that leaves out uncertainties or difficult admissions (such as planning to act in ways that had the potential to cause harm), and that involves ‘after the event’ reconstructions or rationalisations. The interview approach we used may also have played a part in the creation of a particular narrative. We did, however, strive to ensure as much as possible that participants were given the time and space to recount their stories in their own words and in their own way.

It is important to acknowledge, too, that the interviews described here could also be interpreted as confirming that people do experience factors such as ‘increased cognitive rigidity’ and ‘impaired decision-making’ in the sense that planning to end one's life might indeed require a determination that could be read as rigidity, and the fact of the desired or expected outcome being death could be described as an outcome of impaired decision-making. Similarly, going over possible actions in one's mind (‘rehearsing’ or imagining events) could be considered a form of rumination or cognitive compulsion, the function of which is to confer relief.

### Implications

First-person accounts may allow us to better understand how people decide upon a particular method, time and place for a suicide attempt. By giving people free rein to describe in their own words the processes they went through in planning and undertaking a suicide attempt, and by not interpreting such accounts through a lens of deficit and pathology, we can gain important insights into how people come to think about, experience, plan and enact a suicide attempt. These insights, we believe, have implications for how we understand the ‘suicidal process’ and people's planning of suicide attempts.

As an example, informed by current research understandings and conceptualisations, the assessment of suicide risk in a suicidal person may assume the presence of some form of cognitive deficiency or dysfunction such as inflexibility, rigidity or impairment in decision-making. The analysis of the qualitative interviews presented here would challenge such assumptions and cast a different light on the degrees of resourcefulness, careful planning, ability to adapt and imagination present in people's accounts of their suicide attempt on the railway.

The interviews also affirmed the importance of paying attention to the personal meanings involved in suicidal behaviour, and, as such, lend weight to approaches to suicide prevention that emphasise the importance of exploring *with* people their suicidal thinking and its meaning (as in, for example, the Collaborative Assessment and Management of Suicidality^34^).

In terms of suicide prevention more broadly, understanding in greater detail and depth the ways in which people come to plan and enact a suicide attempt using a particular method can inform the development and implementation of approaches such as means restriction interventions. That is, insights into the factors that people draw on when considering and choosing a specific method, and their interaction with that method over time when planning and enacting a suicide attempt, may open up possibilities for influencing behaviour based on dissuasion and reducing cognitive availability, which could be used in addition to physical means restriction measures. The interviews outlined here offer insights into the factors that seemingly attract people to the method/location (quick, lethal, accessible, commonly used method) and also what dissuades them (effects on others, especially the driver; possibility of surviving with injuries; possibility of intervention). The logic, in terms of any messaging or communications strategy aimed at prevention, would be to challenge the ‘attractors’ (because many are misunderstandings or myths) and try to reinforce or amplify the ‘dissuaders’. The participants in the study described changing plans, or even abandoning them altogether, in the light of new information or environmental circumstances (e.g. the presence of children); that adaptability offers hope that people's behaviour can be influenced at any point in the ‘suicidal process’.

Suicide, in the accounts from our participants, is described most often as a way out of profound psychological pain and distress. Ways to alleviate the suffering and pain experienced by people vulnerable to suicide should undoubtedly remain the focus of prevention efforts. However, if we interpret people's accounts of their experiences (clinically and from a research perspective) only through a lens of individual deficit and impairment, our understanding of how people actually come to think about, plan and enact a suicide attempt may be unnecessarily limited.

## Data Availability

The Results section of this article provides illustrative examples of our data which have been anonymised. Owing to the qualitative nature of this study, we cannot make full transcripts, footage or survey data available as these could potentially identify our participants.

## Footnotes

a Samaritans is a registered charity aimed at providing emotional support to anyone in emotional distress, struggling to cope or at risk of suicide throughout the UK and Ireland.

b The quotes used in this section, and elsewhere, are representative of the responses of interviewees, but they do not necessarily accurately depict the reality of a suicide on the railways. For example, in the UK the average lethality rate on the railways is around 80%, whereas attempts on the London underground have a lower fatality rate (Railway Suicide and Crisis Incidents – 2017/2018. BTP, 2018).
